# Prospective Observational Study on Evaluation of Cardiac Dysfunction Induced during the Weaning Process

**DOI:** 10.5005/jp-journals-10071-23106

**Published:** 2019-01

**Authors:** Havaldar Amarja, Krishna Bhuvana, Sampath Sriram

**Affiliations:** 1-3 Department of Critical Care Medicine, St. John's Hospital, Bengaluru, Karnataka, India

**Keywords:** Cardiac dysfunction, Doppler echocardiography, pressure support-positive end-expiratory pressure, Spontaneous breathing trial, Weaning

## Abstract

**Context:**

Weaning induced cardiac dysfunction can occur without underlying heart disease. Changes in intrathoracic pressure, systemic vascular resistance, preload and afterload leading to heart-lung interactions are the possible explanatory mechanisms

**Aims:**

The aim of the current study was whether the assessment and identification of cardiac dysfunction induced during the weaning process could predict the outcome of extubation.

**Settings and design:**

A prospective observational study with convenience sampling method was conducted from May 2015 to April 2016 after institutional ethical committee approval (ref 161/2015).

**Materials and methods:**

Patients over eighteen and planned for extubation were included. Weaning method used was a spontaneous breathing trial (SBT) by pressure support-positive end-expiratory pressure (PS-PEEP). Baseline characteristics, weaning, and echocardiography parameters were collected pre extubation. Post-extubation echocardiographic parameters were collected within six hours as per the protocol. The primary outcome was extubation failure (reintubation within 48 hours). Secondary outcomes were ICU length of stay and ICU mortality.

**Statistical analysis:**

Statistical method used is STATA™ (Version14, College Station TX).

**Results:**

Out of one hundred and sixty-one patients, twenty-one failed extubation (13.04 %). Pre-extubation echocardiographic parameters were similar in two groups except for preexisting LV systolic dysfunction. Post-extubation E/e` (9.30 *vs. *7.71 p = 0.018) was higher in the extubation failure group. Extubation failure group had higher intensive care unit (ICU) length of stay and ICU mortality.

**Conclusion:**

In our study E/e` during a weaning trial did not predict extubation success. Cardiac dysfunction induced during weaning may get masked during weaning and manifests postextubation. This needs to be verified in subsequent studies.

**Key messages:**

Cardiac dysfunction induced during the weaning process may get masked and manifests post-extubation. Echocardiographic assessment during the weaning process and post-extubation helps to evaluate and identify the patients at risk of reintubation.

**How to cite this article:**

Amarja H, Bhuvana K, Sriram S. Prospective Observational Study on Evaluation of Cardiac Dysfunction Induced during the Weaning Process. Indian Journal of Critical Care Medicine, January 2019;23(1):15-19.

## INTRODUCTION

Weaning the patient from ventilator support is crucial, and the process needs to be initiated at the earliest and when there is a resolution of primary pathology.^1,2^ Weaning failure is defined as failure to pass SBT or the need for reintubation within 48 hours following extubation. The incidence of extubation failure is around 2 to 25%.^3,4^ Extubation failure is associated with prolonged ICU stay and mortality hence it's prediction is important.^3^

Cardiac dysfunction induced by weaning can occur without underlying heart disease.^5-8^ The change in intrathoracic pressure during the process of weaning can precipitate cardiac dysfunction.^6-8^ Mechanisms which have been proposed include (a) increased adrenergic tone which results in redistribution of venous blood from the unstressed volume towards the stressed volume; (b) an increase in the systemic venous pressure gradient, central blood volume, and RV preload; (c) changes in intra-thoracic pressure resulting from the transition from positive pressure ventilation to spontaneous breathing, can increase LV preload, afterload, systolic arterial pressure, and heart rate.^5^

In a small trial, Lemaire et al. noted elevation of pulmonary artery occlusion pressure (PAOP) in patients who failed SBT.^9^ The conclusion of this study was not easily generalizable as the study included difficult to wean chronic obstructive pulmonary disease (COPD) patients and the study was performed in only fifteen patients. Similar trials have been done in the past in subgroups of patients such as COPD, patients with neurological disease, without underlying heart disease or patients without arrhythmia and the external validity of such trials is limited. ^10,11^ Recent meta-analysis evaluating diastolic dysfunction as a cause for weaning failure showed higher E/e` was associated with weaning failure and taking into account wide heterogeneity in the criteria for diastolic dysfunction in trials included, these findings could not be easily generalizable.^11^

Conventional weaning parameters such as rapid shallow breathing index (RSBI), a value of less than one hundred and five cannot identify the possibility of extubation failure.^12,13^ The need for parameters which can guide treating physicians in deciding for extubation has been highlighted by studies.^14,15^

The literature on cardiac dysfunction induced by weaning in a general ICU population in Indian settings is scanty. The predominantly weaning method used in ICU's is pressure support-positive end-expiratory pressure (PS with PEEP). During the process of weaning heart, lung interactions can cause cardiac dysfunction, and it affects the outcome of extubation. With an increase in the incidence of cardiac co-morbidities and elderly population the study on cardiac dysfunction induced by weaning is indicated and appropriate. Cardiac dysfunction can be assessed noninvasively by echocardiography at the bedside.^16-19^ The aim of the current study was whether the assessment and identification of cardiac dysfunction induced during the weaning process could predict extubation success in the general ICU population.

## MATERIALS AND METHODS

Formal institutional ethical committee approval was obtained before initiating the study (ref no 161/2015). The study was conducted for a duration of 1 year from May 2015 to April 2016. It was a prospective, observational study conducted in the thirty bedded ICU of a large university medical center. Patients were enrolled and included, after obtaining consent from patients or legally acceptable representatives. All patients above eighteen years of age, who were planned for extubation as per the decision of treating team was included in the study. Tracheostomised and pregnant patients were excluded. Weaning method used in the study was PS with PEEP. After successful completion of SBT by PS with PEEP as per the ICU protocol, patients planned for extubation based on treating physician's decision were included in the study. The convenience sampling method was used for patient selection. Patients with preexisting comorbid illnesses were included in the prespecified subgroups. Comorbidities were classified based on ICD 10 (International Statistical Classification of Diseases). Patients with preexisting cardiac illnesses were also part of our study

All measurements were performed by the first author to avoid interobserver bias. An average of three recordings over five minutes was taken to avoid intra-observer bias. Baseline characteristics, weaning, and echocardiography parameters were collected pre extubation. The extubation was performed immediately after the collection of parameters. Repeat echocardiographic assessment was planned as these patients were followed up till 48 hours and the primary outcome of the study was extubation failure (reintubation within 48 hours). Repeat echocardiography after six hours post-extubation was planned as per the protocol to look for any change in the echocardiographic parameters. All patients were followed until discharge from ICU. Secondary outcomes were mortality and ICU length of stay.

Conventional weaning parameters like respiratory rate, tidal volume, RSBI, FiO_2_, PEEP, PaO_2_/FiO_2_ were collected. ^6^ Acute physiology, age and chronic health evaluation score (APACHE II) was calculated from records of the first twenty-four hours of ICU admission. The cumulative fluid balance of 3 days before extubation was calculated. Hemoglobin on the day of extubation measured on ABG machine was collected. All patients were followed up until ICU discharge. Extubation failure was defined as the requirement of mechanical ventilation support within 48 hrs of extubation.^5^

### Echocardiographic Measurements

All measurements were done using a Sonosite™ Edge portable machine with a 2.5 MHz probe and inbuilt cardiac calculation software. LV systolic function was assessed qualitatively by visual gestalt method taking into account LV size, contractility and thickening of the various segments and classified into good, mild, moderate, moderate to severe and severe LV dysfunction. Qualitative assessment of systolic function was done due to ease of its use. LV diastolic dysfunction was assessed by transmitral flow velocity. Early filling phase (E), late filling phase (A) and deceleration time (DCT) were measured. E/A was calculated. Assessment of mitral annular flow velocity was done using tissue Doppler imaging at the lateral mitral annulus. e` and *a*` were measured. In patients having regional wall motion abnormalities (RWMA), both lateral and septal mitral leaflet velocities were measured and averaged. The right ventricular systolic function was assessed using tricuspid annular plane systolic excursion (TAPSE) and inferior vena cava (IVC) diameter was measured. E/A, E/e` was calculated.

## RESULTS

Statistical methods STATA™ (Version14, College Station TX) was used for statistical analysis. Data were expressed as mean (SD) or median (Interquartile range IQR) as appropriate. Bivariate analysis of primary outcome versus independent characteristics was done using c^2^ test for categorical variables and 't' test (Satterwhite's for unequal variance) for continuous data. 'Mann-Whitney U' test was used for non-parametric continuous variables. A standard 95% confidence limit and a p-value at 0.05 were used for assessing statistical significance.

**Table 1:** Baseline Parameters

*Baseline parameters mean (SD)/ Median (IQR)*	*Extubation success (n = 140)*	*Extubation failure (n = 21)*	*p-value*
Age	47.40 (17.27)	51.85 (22.95)	0.8
Gender (M/F)	74/66	13/8	0.64
APACHE II	18.02 (6.61)	20.85 (5.65)	0.12
Days on ventilator	2 (1-4)	4 (2-5)	0.01*
Cardiac disease	46 (32.85)	11 (52.38)	0.08
Renal disease	20 (14.28)	5 (23.80)	0.26
Respiratory	10 (7.14)	0 (0)	0.20
Endocrine	43 (30.71)	9 (42.85)	0.26
Cumulative fluid balance	1943 (480-3180)	2289 (912-3416)	0.38

*significant p < 0.05

**Table 2:** Pre-extubation echocardiographicassessment

*Pre extubation ECHO Mean (SD)/ Median (IQR)*	*Extubation success (n = 140)*	*Extubation failure (n = 21) p-value*
E (cm/sec)	81.91(19.27)	92.52 (24.94)	0.07
A (cm/sec)	69.95 (19.61)	77.92 (26.39)	0.19
E/A	1.25 (0.47)	1.30 (0.55)	0.72
DCT (ms)	119.22 (38.18)	116.48 (36.04)	0.75
e` (cm/sec)	11.38 (3.24)	11.43 (3.17)	0.95
a` (cm/sec)	9.56 (2.78)	10.16 (2.43)	0.31
E/e`	7.68 (2.79)	8.21(2.95)	0.45
TAPSE (cm/sec)	1.84 (0.53)	1.83 (0.32)	0.95
IVC (cm)	1.6 (0.3)	1.6 (0.4)	0.8
Severe LV dysfunction	9/131	7/14	< 0.001*

*significant p <0.05

The variables identified as significant in the bivariate analysis were used as independent variables and outcomes were used as dependent variables to develop a predictive model using logistic regression. Measurements obtained pre and post-extubation were analyzed using conditional logistic regression.

Total one eighty patients were screened. Nineteen patients were excluded due to poor echo window (10.5%). Out of one sixty-one patients, one forty (86.95%) patients were successfully extubated and twenty-one (13.04%) patients had extubation failure. The summary baseline characteristics, for reintubated and successfully extubated patients, were collected (Table 1). Baseline characteristics were comparable between patients with extubation success and failure group.

The measured echocardiographic parameters were compared pre and post-extubation for both failed and successful extubations (Table 2 and 3). Echocardiographic assessment of systolic function was done by visual gestalt method. Out of 161 patients, 20 (12.42%) had severe LV systolic dysfunction. Diastolic dysfunction by transmitral flow velocity using pulsed wave Doppler (PWD) showed E/Aof 1.25 in successful and E/A 1.30 in failed extubation group. Mitral annular flow velocity by tissue Doppler imaging (TDI) showed pre extubation E/e` was not statistically significant in extubation success and failure group (7.68 *vs. *8.21 p = 0.45). There was no echocardiographic parameter which could be measured pre-extubation which was statistically significant between the two groups except for pre-existing severe LV dysfunction.

**Table 3:** Post-extubation echocardiographic assessment

*Postextubation ECHO Mean (SD)/Median (IQR)*	*Extubation success (n = 140)*	*Extubation failure (n = 21)*	*p-value*
E (cm/sec)	86.04 (20.41)	99.08 (35.68)	0.11
A ( cm/sec)	72.52 ( 20.24)	77.18 (38.27)	0.59
E/A	1.18 (0.91-1.53)	1.20 (0.93-1.75)	0.21
DCT (ms)	121.60 (36.46)	114.98 (32.77)	0.40
e`(cm/sec)	11.71 (3.20)	10.78 (2.83)	0.18
a`(cm/sec)	10.0 (2.72)	9.51 (2.88)	0.46
E/e`	7.71 (2.72)	9.30 (2.72)	0.018*
TAPSE (cm/sec)	1.95 (0.49)	1.70 (0.70)	0.15
IVC (cm)	1.66 (0.52)	1.65 (0.41)	0.9

*significant p <0.05

In the parameters measured post-extubation, E/e` was statistically significant between extubation success and failure group (7.71 *vs. *9.30 p = 0.018) (Table 3). The ventilatory parameters were not statistically significant between the two groups (Table 4). In secondary outcomes, patients in the extubation failure had higher ICU length of stay and mortality (Table 5). Attempts to fit a logistic model did not identify any combination of co-variates (Echo and respiratory) which could predict successful extubation. Similarly, conditional logistic regression could not identify parameters which could predict extubation success.

## DISCUSSION

Echocardiographic assessment before extubation in unselected ICU population is feasible and practical. In our study, there were only 19 patients (10.5%) in which echocardiographic assessment could not be done. As compared to the previous study by Lemaire et al., where PAOP was measured, in our study, cardiac dysfunction was assessed non-invasively by echocardiography.

**Table 4:** Ventilator parameters

*Ventilator parameters*	*Extubation success (n = 140)*	*Extubation failure (n = 21)*	*p-value*
TIDAL VOLUME (mL)	451.69 (118.56)	421.66 (91.81)	0.19
RR	20.09 (5.70)	22.85 (5.96)	0.05
RSBI	68.97 (30.64)	66.41 (23.20)	0.65
PS	8.94 (2.43)	9.57 (3.57)	0.44
PEEP	5.70 (1.01)	6.14 (1.42)	0.18

**Table 5:** Primary and Secondary outcomes

* Primary and Secondary Outcome Mean(SD)/ Median (IQR) *	*Extubation success (n = 140)*	*Extubation failure (n = 21)*	*p-value*
Extubation	140 (86.95%)	21 (13.04%)	
ICU LOS	6 (4-8)	13 (8-19)	<0.001*
ICU mortality	11/129 (7.8%)	11/10 (52.3%)	<0.001*

*significant p <0.05

Previous trials showed E/e` was the echocardiographic parameter which was a good predictor of extubation success. The recent study by Pappanikolaou et al. showed E/e` at lateral mitral leaflet of > 7.8 may identify patients at high risk of weaning failure.^20^ In the study by Moschietto et al, patients were weaned by SBT with PS and showed higher E/e` in extubation failure group. LV systolic dysfunction was not found as a cause for weaning failure.^21^ A recent study also showed isolated diastolic dysfunction was associated with prolonged weaning.^22^ In our study pre extubation assessment E/e`(7.68 *vs. *8.21, p = 0.45) was not found as a statistically significant parameter but pre-existing LV systolic dysfunction was found to be a statistically significant parameter.

The possible reason for the difference in the results of our study as compared to previous is the method used for weaning. SBT with T-piece was the weaning method used in the majority of the previous studies. ^19^ SBT with PS-PEEP helps in overcoming load dependent diastolic heart failure and causes a reduction in LV preload and LV afterload thereby improving LV emptying. As a result of which cardiac dysfunction during weaning gets masked and becomes more evident post-extubation as shown in the present study.^23,24^ In our study, cardiac parameters measured post-extubation showed E/e` was higher in the group who failed extubation (9.30 *vs. *7.71 p = 0.018) and was statistically significant.

Reintubation is associated with increased length of ICU stay and mortality, and it is important to time the extubation appropriately.^2-4^ The current study has shown that cardiac dysfunction possibly precipitated by the withdrawal of pressure support and peep can be picked up by post-extubation echocardiographic assessment.

The limitation of the current study was that all readings were collected by a single observer. The effect of inter-observer variability could not be assessed. Patients who successfully completed SBT were included, and patients were not classified based on simple, difficult and prolonged weaning as extubation was the primary outcome of the study. Days on a ventilator was one of the baseline variables which was statistically different in the two groups, but it was a non-significant covariate in the logistic model. Patients having higher E/e` post-extubation had higher chances of reintubation. For the results of this study to be generalizable, a larger study in which the echo parameters are collected reliably and with sufficient precision by multiple observers and involving repeated echocardiographic assessment is indicated. Different weaning methods and its effect on extubation success need to be evaluated in subsequent studies.

Recent studies have highlighted the importance of the preload condition of the right and left ventricle and the effect of passive leg raising and fluid removal on weaning-induced cardiac dysfunction.^25,26^

Future studies involving pre extubation assessment of Cardia, Lung, Pleura, and Diaphragm, is required to help the clinician in identifying patients at risk of extubation failure due to multiple factors.^27^

## CONCLUSION

In our study, echocardiographic measurements prior to extubation showed preexisting LV systolic dysfunction could predict extubation. A higher E/e` post-extubation was associated with chances of reintubation. A larger study is indicated in developing a predictive model for extubation from echo parameters.
